# The Assessment of Dermatology Clinical Research in Saudi Arabia

**DOI:** 10.7759/cureus.15879

**Published:** 2021-06-23

**Authors:** Mawaddah A Tallab, Sarah B Aljoudi, Sultan S Alfaer, Fedaa S Andijani, Jehad O Hariri, Mohammed H Abduljabbar

**Affiliations:** 1 Department of Dermatology, King Fahad Military Medical Complex, Jeddah, SAU; 2 Department of Dermatology, King Abdulaziz University Hospital, Jeddah, SAU; 3 Department of Dermatology, King Fahad General Hospital, Jeddah, SAU

**Keywords:** level of evidence, research in dermatology, clinical research, research in saudi arabia, evidence-based medicine

## Abstract

Objectives: To determine the level of evidence in dermatology research over the last five years and to assess the frequency of publication in different journals in the field of dermatology in the kingdom of Saudi Arabia, western region.

Methods: All published research were reviewed during the period of 2015 till 2020 using online research database through PubMed, Embase, and Google Scholar. A list of all Saudi dermatologists who are registered by the Saudi Commission for Health Specialties as consultants, and who worked in public institutions at Jeddah and Makkah was retrieved. The Oxford Level of Evidence Scale was utilized to determine the level of evidence of these studies. Descriptive statistics were used to determine the frequency of different study types and levels of evidence.

Results: A total of 125 articles were published in 62 different national and international journals. Majority of the published studies were level IV (76%). Case reports were the most common type of published research (56%) and meta-analysis studies accounted for (6.4%). Thirty-two articles were produced by academic institutions, compared to 68 published articles from governmental institutions, and 22 from military hospitals.

Conclusion: Only a small percentage of publications in Saudi Arabia are considered high level clinical research. The number of publications during the past five years was high compared to the previous years and case reports constituted the majority. Authors should be encouraged to conduct higher-level studies to enhance patient care.

## Introduction and background

Nowadays, evidence-based medicine is considered one of the essential tools used in clinical practice. It helps the physician reach standardization in decision making and medical care [1]. In dermatology practice, reliance on research is essential as it is a rapidly growing medical science. Evidence-based dermatology aids in being systematic and explicit, keeping up to date with increasing precision and minimizing bias [2].

However, the cornerstone in evidence-based medicine is the sequence of evidence from medical research as the number of valid evidence has been increasing in the last decade. Many classification tools had been proposed in the literature [3,4]. The Oxford Centre for Evidence-Based Medicine in 1998 classified the level of evidence into five categorical classes based on data retrieved from multiple studies. The classification used in this study helps sort out publications based on this classification. The level of evidence described ranges from level I which represents randomized controlled trials and meta-analysis, to level V which represents an expert opinion [5].

In Saudi Arabia, several healthcare institutes, as well as medical schools, have been contributing largely to the development of research in dermatology. It is necessary to assimilate, evaluate and appraise each piece of evidence for the best interest of our patients and hence this study.

The aim of this study is to determine the level of evidence in dermatology research over the past five years. Our secondary aim is to assess the frequency of publications from Saudi Arabia in different dermatology journals.

## Review

Methodology

**Figure 1:**
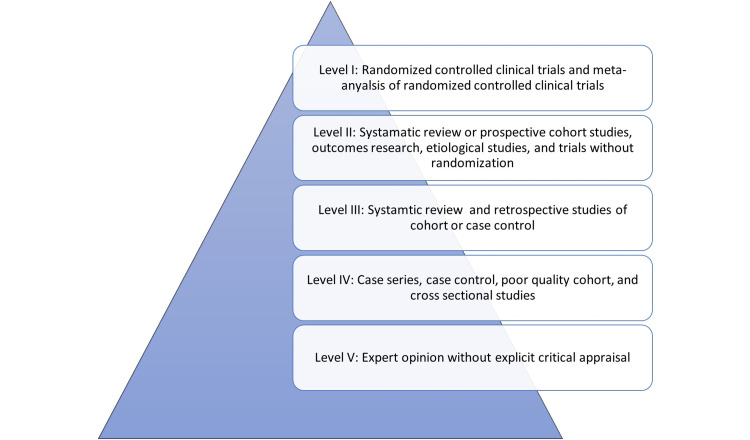
Oxford Centre for Evidence-Based Medicine Levels of Evidence Scale

All published research was reviewed during the period of 2015 till 2020 using online research database through PubMed, Embase, and google scholar. A list of all Saudi dermatologists who are registered by Saudi Commission for Health Specialties as consultants, and who worked in public institutions at Jeddah and Makkah was retrieved. These institutions include all academic universities (King Abdul-Aziz University and the University of Jeddah), military institutions (National Guard Hospital and King Fahad Armed Forces Hospital), governmental institutions (King Fahad General Hospital, East Jeddah Hospital, King Abdullah Medical Complex, Hera’s General Hospital, and King Faisal Hospital), and main hospitals (King Faisal Specialist Hospital and Research Center). Inclusion criteria were studies published in English by Saudi dermatologists in Jeddah and Makkah in the previously mentioned institution in national and international journals during the past five years periods. Studies were categorized into eight main categories: (1) randomized control trial, (2) meta-analysis, (3) prospective cohort, (4) retrospective cohort, (5) cross-sectional studies, (6) epidemiological studies, (7) case series, and (8) case reports. Letters to the editor and non-human studies were excluded. The Oxford Level of Evidence Scale was utilized to determine the level of evidence of these studies. Studies were ranked according to the levels: Level I (highest evidence, for example, meta-analysis of randomized control trials) to Level V (lowest evidence, for example, basic science) (Figure 1).

Statistical analysis

Statistical analysis was performed using STATA 16.1 (STATA Corp., College Station, TX, USA). Descriptive statistics were used to determine the frequency of different study types and levels of evidence, along with percentages. A chi-square test was used to compare categorical variables.

Results

**Figure 2:**
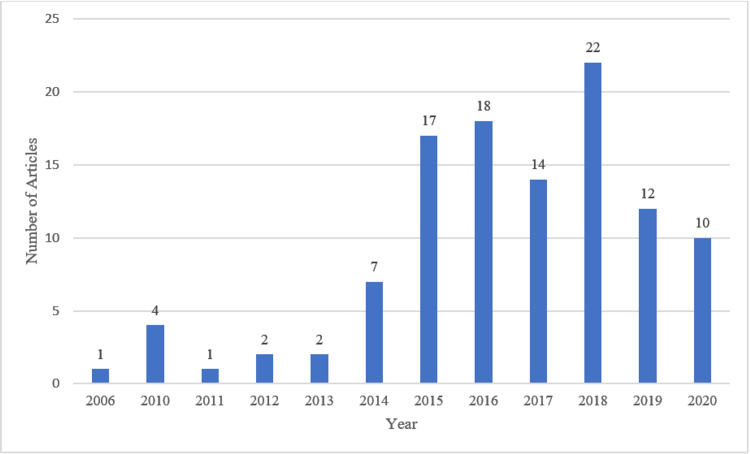
A Graphic Demonstration of Number of Saudi Dermatology Publications per Year

One hundred and twenty-five articles were published in 62 different national and international journals (Table 1). The number of yearly publications during the period 2015 to 2020 was higher compared to the period from 2006 to 2014. During the period from 2015 to 2020, the annual publications were as many as 23 (20%), while from 2006 to 2014, there were only one to seven publications per year (Figure 2).

**Table 1:** Frequency of Publications in Different Journals in the Field of Dermatology

Journal	Frequency of Publication	Percentage (%)
Journal of Dermatology and Dermatological Surgery	11	(10)
Journal of Health Science	8	(7.27)
Case Reports in Dermatology	6	(5.45)
Journal of the Saudi Society of Dermatology and Dermatologic Surgery	5	(4.55)
International Journal of Medical Research Professionals	4	(3.64)
The Open Dermatology Journal	4	(3.64)
Acta Dermato-Venereoligica	3	(2.73)
International Journal of Research in Dermatology	3	(2.73)
Journal of Family Medicine and Primary Care	3	(2.73)
Journal of Medical Science	3	(2.73)
Journal of the European Academy of Dermatology and Venereology	3	(2.73)
Others	57	(51.82)

Level IV studies made up (76%) of the total publications, followed by level III (13.6), level I (7.2%), and level II (3.2%) (Table 2). Case reports were the most common type of published research (56%), followed by cross-sectional studies (16%), retrospective studies (14.4%), and meta-analysis studies (6.4%). Three prospective and four case series studies were found (2.4%, 3.2%), respectively. Two epidemiological studies (1.6%) was observed. Table 3 shows the distribution of articles by type of studies, main subject, and journal type.

**Table 2:** Level of Evidence of Saudi Dermatology Research

Level of Evidence	Frequency	Percentage (%)
Level I	9	(7.2)
Level II	4	(3.2)
Level III	17	(13.6)
Level IV	95	(76)
Total	125	(100)

**Table 3:** Distribution of Articles by Type of Studies, Main Subject, and Journal Type

Type of Studies	Frequency	Percentage (%)
Epidemiological /RCT	2	(1.6)
Meta-analysis	8	(6.4)
Cross-sectional	20	(16)
Retrospective	18	(14.4)
Prospective	3	(2.4)
Case series	4	(3.2)
Case reports	70	(56)
Subject		
General dermatology	66	(52.8)
Oncology	17	(13.6)
Pediatric dermatology	16	(12.8)
Cosmetics	7	(5.6)
Dermatological surgery	6	(4.8)
Dermatopathology	2	(1.6)
Pharmacology	2	(1.6)
Hair	9	(7.2)
Types of Journals		
Saudi journal	23	(18.4)
International journal	102	(82.6)

When analyzing the level of evidence per institution, 32 articles were produced by academic institutions, 27.4% were level IV, and 44.4% were level I. Out of the published articles from governmental institutions (n=68), 55.9% were level IV, and 11.1% were level I. Finally, military institutions (n=22), had 13.7% level IV and 44.4% level I papers out of their total publication output (Table 4).

**Table 4:** Saudi Institutions and Their Contribution to Dermatology Literature

Institution	n (%)	Level of Evidence			
		Level I	Level II	Level III	Level IV
King Abdulaziz University	26(20.8)	1(11.1%)	1(25%)	1(5.9%)	23(24.2%)
Heraa General Hospital	15(12)	0	1(25%)	4(23.5%)	10(10.6%)
National Guard Hospital	13(10.4)	4(44.4%)	2(50%)	1(5.9%)	6(6.3%)
King Fahad Armed Forces Hospital	9(7.2)	0	0	2(11.8%)	7(7.4%)
University of Jeddah	6(4.8)	3 (33.3%)	0	0	3(3.2%)
East Jeddah General Hospital	7(5.6)	0	0	4(23.5%)	3(3.2%)
King Fahad General Hospital	2(1.6)	0	0	2(11.8%)	0
King Abdullah Medical Complex	4(3.2)	0	0	1(5.9%)	3(3.2%)
King Abdul-Aziz Hospital	40(32)	1 (11.1)	0	2(11.8%)	37(38.9%)
King Faisal Specialist Hospital and Research Center	3(2.4)	0	0	0	3(3.2%)
Total	125(100)	9(100%)	4(100%)	17(100%)	95(100%)

Discussion

There is a paucity of high-level evidence research as seen by the low number of level I (7.2%) and II (3.2%) studies in Saudi Arabia. Most of the publications were at level IV (76%). Upon comparison with other similar local studies that share the same methodology, we found that level IV evidence is seen in 91% of published research in neurology and plastic surgery, 86% of orthopedic, 49.5% of abdominal surgery [6-9]. However, all previous mentioned studies demonstrated weakness in publishing research with high level of evidence. We think that one of the reasons that studies conducted in Saudi Arabia are positioned lower on the evidence scale is the issue of lacking a Saudi national registry which could lead to lengthening the time needed for data collection and affect the quality of research.

Globally, high level of evidence-based dermatology is well established. The European Dermatology Forum, British Association of Dermatologists, and American Academy of Dermatology have published many clinical practice guidelines based on randomized controlled trials and meta-analysis (level I) [10,11]. 

The average number of publications over the last five years is considered low compared to international figures [12,13]. However, in our obtained data, physicians with university and military affiliations reported the highest number of level I publications compared to others. Reasons behind this could be due to the presence of interest secondary to financial incentives and academic rewards. In contrast, the percentage of level I publications from all institutions fell below 5% since conducting randomized controlled trials or meta-analysis is not always feasible due to multifactorial issues related to funding and/or logistics. In addition, not all physicians are trained for this type of research methodology. Additionally, difficulties in enrolment in clinical trials can be challenging for the population in Saudi Arabia due to cultural issues and unfamiliarity of the society to the process of clinical trials.

As with other specialties, the quality of published randomized controlled trials in dermatology literature are of low quality. Adetugbo and Williams [14] reported difficulties in performing such studies with a large sample size due to the rarity of some diseases in dermatology which enhances type 2 errors. In addition, incomplete reporting of methodological and design flaws in the majority of randomized controlled trials such as improper randomization is a common practice noted in the literature.

Accordingly, we advocate researchers to focus on constructing good methodology designs beside high level of evidence studies to provide the patients with the best care available.

In the authors' point of view, it is important for dermatologists to be aware of each study recommendations based not only on high level of evidence but also good study methodology.

To overcome these boundaries, it is necessary to establish proper research teaching courses and activities in our medical school curriculum and during residency programs [15]. Local dermatology societies should prioritize the need for conduction good-quality randomized trials and other studies to improve the quality of evidence-based medicine in dermatology by funding these projects, provide incentives and prestigious awards. 

The Journal of Dermatology and Dermatological Surgery was the most frequently used site for publications. Most of the articles were published in international journals with only 18.4% published in Saudi journals, which is considered the lowest compared to what others have been citing for publications in local journals [7,8]. General dermatology was the most reported subject, where dermatological surgery and the field of cosmetics were the least. In the authors’ opinion, this reflects the general practice of the dermatologist in Saudi Arabia in the western region in which the surgical and cosmetic fields are not frequently practiced in governmental institutions and extremely limited in military and academic hospitals. In addition, technical aspects in surgery make it exceedingly difficult to design study research. Finally, the most common reported type of study were case reports followed by cross-sectional studies consistent with results reported by Maghrabi et.al. [6] and Jamjoom et.al. [8]. The popularity of case reports comes from the fact that they are easy to perform in a short time and no financial support is needed [16]. It is more popular in the field of dermatology due to rarity of diseases and highly updated nature of this specialty. 

Study limitation

The lack of studies with similar methodology in the same specialty made it difficult for us to compare and make generalizations. Another limitation includes the misspelling of physicians’ names which results in a decreased captured rate of published studies. Additionally, our study was conducted in the western region and not generalizable toward other regions of Saudi Arabia. The rate of citations for the selected articles was not studied. However, we suggest for future articles to study the frequency of citations of studies done in the western region of Saudi Arabia.

## Conclusions

A small percentage of publications in Saudi Arabia were considered high-level clinical research. Although level IV studies can provide valuable information, especially for rare conditions, authors should be encouraged to conduct higher-level studies as bias and confounding factors can be eliminated and clinical research with low level of evidence could negatively affect patient care. More studies that are high in quality need to be performed in the field of dermatology in Saudi Arabia and collaborative efforts between centers and institutions are essential in improving the evidence that support clinical practice tailored to Saudi society.
